# Possible mechanisms of cholesterol elevation aggravating COVID-19

**DOI:** 10.7150/ijms.62021

**Published:** 2021-08-21

**Authors:** Yan Tang, Longtai Hu, Yi Liu, Bangyi Zhou, Xiaohuan Qin, Jujian Ye, Maoze Shen, Zhijian Wu, Peidong Zhang

**Affiliations:** 1 Department of Cardiology, Heart Center, Zhujiang Hospital, Southern Medical University, 235 Industrial Avenue, Guangzhou, 510282, Guangdong, People's Republic of China.; 2 Department of Cardiology, Raoping County People's Hospital, 161 Caichang Street, Huanggang Town, Chaozhou, 515700, Guangdong, People's Republic of China.; 3 Department of Cardiology, Affiliated Boai Hospital of Zhongshan, Southern Medical University, No. 6, Chenggui Road, East District, Zhongshan, 528403, Guangdong, People's Republic of China.; 4 School of Traditional Chinese Medicine, Southern Medical University, No. 6, Chenggui Road, East District, Zhongshan, 528403, Guangdong, People's Republic of China.; 5 Zhujiang Hospital, Southern Medical University/The Second School of Clinical Medicine, Southern Medical University, No. 6, Chenggui Road, East District, Zhongshan, 528403, Guangdong, People's Republic of China.; UGS: undergraduate students; MD: medical doctor.

**Keywords:** cholesterol, LDL-C, COVID-19, membrane fusion, lipid-lowering drugs

## Abstract

**Importance:** Despite the availability of a vaccine against the severe acute respiratory syndrome-coronavirus-2 (SARS-CoV-2), humans will have to live with this virus and the after-effects of the coronavirus disease 2019 (COVID-19) infection for a long time. Cholesterol plays an important role in the infection and prognosis of SARS-CoV-2, and the study of its mechanism is of great significance not only for the treatment of COVID-19 but also for research on generic antiviral drugs.

**Observations:** Cholesterol promotes the development of atherosclerosis by activating NLR family pyrin domain containing 3 (NLRP3), and the resulting inflammatory environment indirectly contributes to COVID-19 infection and subsequent deterioration. In *in vitro* studies, membrane cholesterol increased the number of viral entry sites on the host cell membrane and the number of angiotensin-converting enzyme 2 (ACE2) receptors in the membrane fusion site. Previous studies have shown that the fusion protein of the virus interacts with cholesterol, and the spike protein of SARS-CoV-2 also requires cholesterol to enter the host cells. Cholesterol in blood interacts with the spike protein to promote the entry of spike cells, wherein the scavenger receptor class B type 1 (SR-B1) plays an important role. Because of the cardiovascular protective effects of lipid-lowering therapy and the additional anti-inflammatory effects of lipid-lowering drugs, it is currently recommended to continue lipid-lowering therapy for patients with COVID-19, but the safety of extremely low LDL-C is questionable.

**Conclusions and Relevance:** Cholesterol can indirectly increase the susceptibility of patients to SARS-CoV-2 and increase the risk of death from COVID-19, which are mediated by NLRP3 and atherosclerotic plaques, respectively. Cholesterol present in the host cell membrane, virus, and blood may also directly participate in the virus cell entry process, but the specific mechanism still needs further study. Patients with COVID-19 are recommended to continue lipid-lowering therapy.

## Introduction

The ongoing coronavirus disease 2019 (COVID-19) pandemic caused by severe acute respiratory syndrome coronavirus 2 (SARS-CoV-2) poses a huge challenge to the global medical system 1. According to WHO, as of 16:22 (Central European Time) on February 18, 2021, the worldwide confirmed cases of COVID-19 were 109,594,835, including 2,424,060 deaths. Although COVID-19 vaccine research has rapidly advanced in the past few months, a number of scientific concerns such as optimization of vaccination regimens, enhanced doses, relevance of protection, vaccine effectiveness, and safety and enhanced surveillance still need addressing to improve vaccine efficacy 2. It appears that SARS-CoV-2 will continue to coexist with humans for a long time. Therefore, further research into the mechanisms of blocking viruses from infecting host cells is critically important.

Studies have shown that patients with underlying cardiovascular disease (CVD) are more susceptible to develop severe COVID-19 or even die from it 3. Cholesterol is one of the risk factors for CVD, and CVD may reflect the indirect mechanism between elevated cholesterol and increased susceptibility to COVID-19. In addition, cholesterol is directly related to the infection mechanism of coronavirus, via membrane fusion, endocytosis, migration, and other processes. In this article, we review both the indirect and direct mechanisms of cholesterol elevation that seem to aggravate COVID-19, in which we find that the link between cholesterol and cell entry 4, and the pathways of viral entry into cells being interesting subjects for targeted therapy of SARS‐CoV‐2 5.

## Cholesterol increases susceptibility to COVID-19 by causing atherosclerosis

Increased cholesterol levels are associated with high CVD risk in both old 6 and young 7 adults, and a confirmed diagnosis of CVD is a prognostic factor for both mortality and severity in COVID‑19 8. Therefore, elevated cholesterol may indirectly aggravate the infection severity in COVID-19, and possibly even mortality, by causing cardiovascular disease.

### Inflammation may play an important role in CVD and COVID-19

Cardiovascular disease is an umbrella term for conditions that affect the heart or blood vessels. The Framingham Heart Study is one of the original epidemiological observations of the relationship between elevated blood cholesterol levels and future atherosclerotic cardiovascular disease (ASCVD) 9. Cholesterol levels play an important role in the development and regression of atherosclerosis. A hallmark of ASCVD is the accumulation of cholesterol in arterial macrophages 10. During the development of atherosclerotic plaques, cholesterol content positively correlates with markers of plaque vulnerability, and negatively correlates with stability markers 11. To further clarify the process of plaque rupture and/or erosion, which is considered a major cause of cardiovascular events, a study showed that during cholesterol crystallization, the volume of the plaque increases rapidly and the sharp crystals pass through the biofilm of vascular epithelial cells in their path 12. Saturation of free cholesterol can result in cholesterol crystallization, which is proven to be able to induce inflammation and instability of atherosclerotic plaques in arteries 13. Cholesterol crystals (CCs) or oxidized low-density lipoprotein (ox-LDL) can be engulfed by macrophages to form CCs in the phagolysosome 14. Recent studies have shown that free cholesterol may form metastable structures that evolve into persistent flat plate CCs and are prone to inflammation and traumatic injury 15.

An autopsy study showed a higher density of macrophages and dendritic cells in atherosclerotic plaques in patients with systemic infection; greater infiltration of macrophages and T-cell lymphocytes in the adventitia of coronary arteries; and more periadventitial fat in deceased patients than those who did not die from infection 16. SARS-CoV-2 itself is an infectious factor, which, like other infectious factors, increases plaque instability, thereby increasing the risk of cardiovascular events and, to some extent, mortality in COVID-19 patients. Moreover, we noticed that inflammation may play an important role in CVD and COVID-19. Inflammation is an important component of atherosclerosis 17, 18 and a cytokine storm is responsible for the severity of COVID-19 and also an important cause of COVID-19-related death. Further, the impaired acquired immune response and the uncontrolled innate inflammatory response may be related to the cytokine storm mechanism in COVID-19 19.

### NLR family pyrin domain containing 3 (NLRP3) may mediate the process of cholesterol aggravation of COVID-19

Members of the interleukin (IL) family can be expressed and/or affected by all cells of the innate immune system; of these, IL-1 is a central mediator of innate immunity and inflammation 20. The NLRP3 inflammasome is an innate immune signaling complex that has been shown to be a key mediator in the production of IL-1 cytokines in atherosclerosis. In atherosclerotic lesions, ox-LDL, CCs, and other rich endogenous danger signals can activate NLRP3 14.

Interestingly, acute respiratory distress syndrome (ARDS) was observed as a characteristic feature of the rapid progression of severe lung injury among those that died from severe COVID-19 infection, and one of its pathological features is activation of the NLRP3 inflammatory pathway 21. In another review, patients with reduced immune fitness may exhibit inactivity of NLRP3 inflammasomes, leading to severe COVID-19 tissue damage and cytokine storm 22. Furthermore, some researchers hypothesize that excessive activation of the NLRP3 inflammasome may be one of the main factors leading to serious complications in COVID-19 in patients with underlying uncontrolled diabetes 23. A similar conclusion was reported in another review on obesity: the initial deficiency in defense mechanisms that was reflected in the increased susceptibility to SARS-CoV-2 in obesity-related metabolic disorders is most likely due to elevated systemic metabolic inflammation 24, which is closely related to the NLRP3 inflammasome.

**Figure 1:**
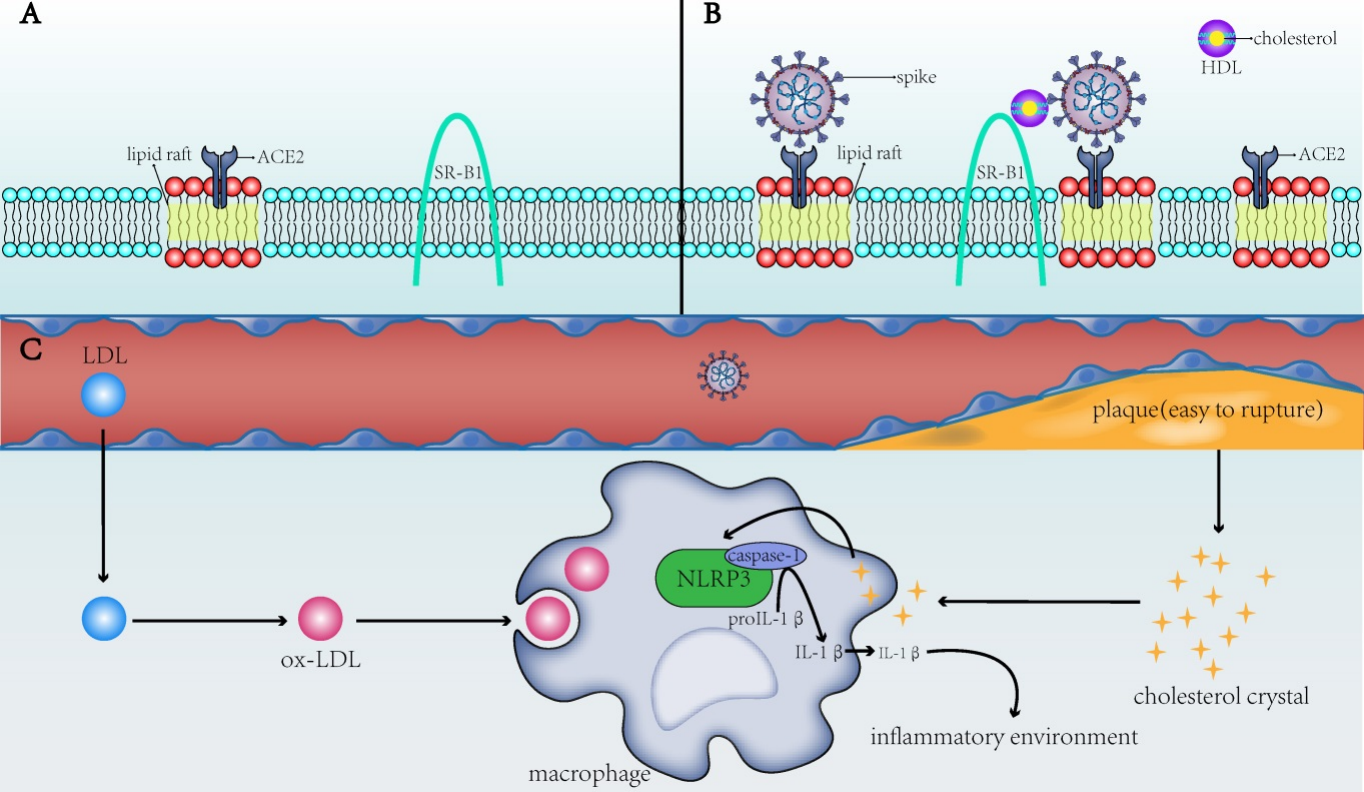
** The direct and indirect mechanisms by which cholesterol affects the infectivity of SARS-CoV-2 and the severity of COVID-19. A)** ACE2 is the receptor for SARS-CoV-2 to enter the host cell and is present in the lipid rafts of the cell membrane; SR-B1 is the membrane receptor of HDL. **B)** When the membrane cholesterol increases, the number of lipid rafts on the cell membrane and on the ACE2 receptor in the lipid rafts also increases. SR-B1 mediates the interaction of cholesterol with the spike of SARS-CoV-2, thereby facilitating the entry of SARS-CoV-2 into the cell. **C)** When blood cholesterol levels increase, the formed cholesterol crystals will make the plaques unstable and prone to rupture, increasing risks to the thromboembolism in patients. After the CCs and ox-LDL are endocytosed by macrophages into the cells, they activate the NLRP3 inflammasome to produce interleukins (e.g., IL-1β) and create a favorable inflammatory environment for viruses to invade cells. SARS-CoV-2: severe acute respiratory syndrome-coronavirus-2; ACE2: angiotensin-converting enzyme 2; SR-B1: scavenger receptor B type 1; HDL: high-density lipoprotein; ox-LDL: oxidized low-density lipoprotein; NLRP3: NLR family pyrin domain containing 3; IL-1β: interleukin-1 beta.

To summarize, ox-LDL and CCs in macrophages of patients with CVD activate the NLRP3 inflammasome to promote the development of atherosclerosis, which, in turn, induces a systemic inflammatory state that provides a favorable scenario for infection with SARS‐CoV‐2 (**Figure 1C**). This not only partially explains the susceptibility to SARS-CoV-2 in patients with underlying CVD but also explains the dysregulated lipid metabolism in patients with a history of SARS-CoV infection 25. The activated NLRP3 in patients with SARS-CoV can contribute to vascular injury and even CVD after recovery from SARS-CoV. In COVID-19, the chronic damage to the cardiovascular system 26, which is similar to that due to SARS-CoV, may also be found; therefore, cardiovascular protection should be paid attention to in the treatment of COVID-19 27. NLRP3 is not the only pathway through which atherosclerosis and COVID-19 interact. Other possible associations remain 28, but NLRP3 is a good explanation for cholesterol's role in COVID-19 susceptibility.

## Cholesterol affects susceptibility by influencing the process of membrane fusion

The entry of nucleocapsid into the host cell is the first step in coronavirus infection, which in turn depends on the fusion of their envelope with the host cell membrane 29. The glycoproteins in the virus spikes (S) mediate the entry of the coronavirus 30. The spike has two subunits - S1 and S2 31 - which mediate receptor binding and membrane fusion, respectively 1. Further, other studies have supported the important role of cholesterol engagement in cell entry 32. Interestingly, membrane cholesterol has been shown to alter the oligomer status of membrane-bound peptides and the effect of altered peptide binding on depth-dependent membrane tissue and kinetics 33. Cholesterol is ubiquitous in the plasma membrane of eukaryotes 34, so this could provide a potential target against coronavirus infection.

### Cholesterol increases the density of ACE2 receptors on host cell membranes

Cholesterol is an important component of lipid rafts. Cholesterol can fill the gap between the associated sphingolipids, resulting in a tighter alignment of cholesterol and sphingolipids, which makes the lipid raft more resistant to washing than the non-lipid raft. The binding of cholesterol and saturated lipids promotes the formation of lipid rafts, which are relatively dense membrane domains and have a higher affinity for certain proteins and lipids 35. A study showed that the ACE2 receptor which is on the host cell membrane mediates the entry of SARS-CoV-2, and then the transmembrane serine protease transmembrane protease, serine 2 (TMPRSS2) activates the S protein 36, while ACE2 co-localizes with cholesterol and sphingolipid-rich lipid raft microdomains in the plasma membrane of pneumocytes 37. The role of lipid rafts in cell entry has been summarized in a previous review, which reported that although many studies have analyzed the influence of cholesterol consumption on viral entry, cholesterol dependence does not necessarily imply involvement of membrane rafts 38. Hence, more research is required to verify these findings.

One study on SARS-CoV suggested that cholesterol-rich microdomains provide a platform for promoting effective interaction of S protein with cellular receptor ACE2 39. Wang et al. found that using cholesterol from serum to increase the cholesterol in cell membranes could enhance the invasion of pseudo-SARS-CoV-2 and the ability of the virus to infect, and both the number and apparent diameter of monosialotetrahexosylganglioside (GM1) lipid rafts (the number of viral entry points of ACE2) on cholesterol-loaded cells increased 40. Wang et al. also found that cholesterol can simultaneously transport ACE2 to the endocytic entry point that the virus might dock, thus effectively utilizing it to enter cells 40. In other words, cholesterol may increase the entry of pseudo-SARS-CoV-2 by increasing the number of endocytic entry points and transporting ACE2 to the endocytic entry point (**Figure 1A,B**). It should be noted that this observation is only from an *in vitro* study and cannot fully reflect the actual situation.

### Cholesterol in the virus may interact with the fusion protein

A lot of current research has focused on cholesterol dependence during membrane fusion, but it should also be considered whether cholesterol binds to the proteins involved in this process. The cholesterol recognition/interaction amino acid consensus (CRAC) domain is one of proteins' structural properties that makes proteins preferentially bind to those high cholesterol domains 41. The role of CRAC in fusion protein gp41 of human immunodeficiency virus (HIV) has been extensively studied 42-44, and the CRAC motif in a flexible domain of the outer membrane domain prior to the transmembrane segment determines the binding of gp41 to cholesterol 45. Interestingly, in a study of the primary sequence of SARS-2-S, some CRAC sequences and its inverted sequence CARC were also found 46. In addition, the entry of HIV-1 requires not only envelope glycoproteins to be redistributed into clusters but also cholesterol in the viral membrane. A residue in the cytoplasmic tail of gp41 directly interacts with cholesterol in the viral cell membrane 47. There is evidence that the C-terminal of the spike's cytoplasmic tail of SARS-CoV-2 is the key to retention of membrane-mediated spike in the endoplasmic reticulum-Golgi intermediate compartment (ERGIC) 48.

Ebola fusion glycoprotein (GP) is associated with disengagement of infected cells, ultimately leading to leaky blood vessels and hemorrhagic fever. The results show that cholesterol and transmembrane domains mediate full-length GP1, 2, or GP2-induced detachment 49. A study conducted on Ebola virus showed that the detachment induced by full-length GP1, 2, or subunit GP 2 depends on cholesterol and the structure of the transmembrane domain^50^. Transmembrane domains (TMDs) of viral fusion proteins or amino acid residues adjacent to TMDs may be involved in the cell fusion process of viruses 51, and the trimerization process of the SARS-CoV-2 spike glycoprotein is induced by the fusion of TMDs and the receptor binding domain 52. The similarity between SARS-CoV-2 and SARS-CoV is that their TMD is very conservative, and both are essential for membrane fusion and intercellular fusion activity 52.

Currently, there are relatively few studies on the role and molecular mechanism of the spike and cholesterol in SARS-CoV-2. However, it is of great significance to further elucidate this mechanism in order to reduce coronavirus infection and drug development. A recent study has shown that the spike requires membrane cholesterol without the need for raft engagement 4. Based on this, future in-depth studies should be conducted.

## HDL and SR-B1 play a role in the infection process

Patients with COVID-19 have significant dyslipidemia, which is closely related to cardiovascular disease. The concentration of HDL, LDL-C, and total cholesterol (TC) in COVID-19 patients was significantly reduced. HDL is significantly decreased in critically ill patients, while LDL and TC are decreased but still dominant 53, indicating an increased risk of cardiovascular disease in COVID-19 patients. Patients with COVID-19 are more prone to systemic inflammatory responses and cytokine storms 54. Studies have shown that patients with diseases such as systemic lupus erythematosus and HIV infection, which are associated with inflammation, have significantly lower levels of HDL than other patients 55, 56. Inflammation seems to cause a decrease in HDL levels, although the mechanism by which HDL levels are reduced is still not clear. Therefore, dyslipidemia that is caused by SARS-CoV-2 infection may be related to the systemic inflammatory response caused by COVID-19. The main function of HDL is to promote the reverse transport of cholesterol from the liver to the periphery, and it also has immunomodulatory, antithrombotic, and antioxidant effects 57. When HDL antioxidant function is impaired, it causes lipid oxidation, which worsens the body's inflammatory response. An antioxidant enzyme in HDL - paraoxonase-1 (PON1) - is also inactivated in response to inflammation-induced oxidative stress, which further impairs HDL function 58. PON1 activity is related to the prognosis of CVD as described above, and low PON1 activity leads to poor prognosis of CVD 59, 60.

SARS-CoV-2 binds to ACE2 through the C-terminal domain of S1 subunit (RBD) 61. Wei et al.^62^ found that HDL-scavenger receptor B type 1 (SR-B1) can help S1 subunit to bind to cholesterol, especially HDL, and help SARS-CoV-2 to enter into the host cells (**Figure 1A,B**). Inhibiting the function of SR-B1 or blocking the cholesterol binding site of the S1 subunit can inhibit SARS-CoV-2 infection. One of the main functions of SR-B1 is to promote the transfer of cholesteryl esters (CEs) from HDL or other lipoproteins to cells through selective uptake mechanisms, and SR-B1 has been shown to be a key receptor for HCV (hepatitis C virus) entry into the body 63. Additionally, the expression of SR-B1 protein in the liver of mice fed a high fat and high sugar diet was 1.2-1.3 times higher than that of normal mice, suggesting that the high cholesterol level caused by chronic human diseases may promote the overexpression of SR-B1, thereby promoting SARS-CoV-2 infection.

## Inspiration for treatment

Large amounts of clinical data have shown that COVID-19 patients had abnormal cholesterol levels after infection 64, 65. Further results have shown that patients with cardiovascular disease and other underlying conditions are more likely to suffer from SARS-CoV-2, which leads to worsening of the patient's condition and poor prognosis 66, 67. Therefore, controlling the changes in cholesterol in the body may affect the development of COVID-19 disease.

### The cut-offs at a normal blood test of the lipid balance for each component

Blood lipids are the lipids in the blood. Blood tests usually include total cholesterol (TC), low-density lipoprotein cholesterol (LDL-C), high-density lipoprotein cholesterol (HDL-C), and triglycerides (TG). It is generally believed that the critical value of each component is shown in **Table 1**, which refers to the standards of the Mayo Clinic.

It is noteworthy that the critical values ​​shown in **Table 1** are only approximate values 68, which are not exactly the same in the guidelines of different regions 69, 70. In the United States, cholesterol levels are measured in milligrams per deciliter (mg/dL), while in Canada and many European countries it is millimoles per liter (mmol/L). Normal concentration of each lipid component in plasma contribute to the low risk of cardiovascular diseases. In fact, the target value of blood lipid is not always the same among people of different ages, genders, and cardiovascular risks. It is generally believed that total cholesterol should be less than 200 mg/dL (5.18 mmol/L) and HDL-C should be higher than 60 mg/dL (1.5 mmol/L). The situation with LDL-C is a little more complicated. Lowering LDL-C is one of the few established and proven principles for the prevention and treatment of atherosclerosis 71, so the guidelines regard it as the goal of lowering lipids. According to different cardiovascular risk levels, patients are divided into low-risk, medium-risk, high-risk, and extremely high-risk 70. Current guidelines recommend the lowering of LDL-C to 115 mg/dl (3 mmol/l) in patients with low and moderate risk; the LDL-C treatment target is <100 mg/dl (2.6 mmol/l) for patients at high risk and <70 mg/dl (1.8 mmol/l) for patients at very high risk 71. TG is not used as a strong indicator, because studies have shown that the causal influence of TG-rich lipoproteins and their residues on ASCVD risk depends on the circulating concentration of ApoB particles, rather than the TG content itself 68, 72.

### LDL-C-lowering therapies

Whether it is for COVID-19 patients or non-COVID-19 people, it is beneficial to maintain the LDL-C in the blood within the normal range. On the one hand, it can reduce the population's susceptibility to COVID-19, and on the other hand, it can also reduce the cardiovascular complications of COVID-19 patients. LDL-C is the main indicator for lowering blood lipids. Lipid-lowering treatment mainly includes lifestyle intervention and drug treatment. For patients who have difficulty in lowering blood lipids, lipid apheresis technology is also available.

#### Lifestyle intervention for lowering lipids

Lifestyle factors that can be intervened to improve overall lipoprotein distribution include weight control 73, physical exercise 74, dietary fat control, reasonable intake of dietary carbohydrates and fiber, smoking cessation and alcohol restriction. Studies have shown that when weight loss is 5 to 8 kg, HDL-C increases by 2-3 mg/dL, and LDL-C decreases by 2-5 mg/dL 75. In addition, adding three weekly aerobic and resistance-training sessions to the dieting program may deliver better outcomes 76. Studies have also shown that physical exercise reduced the atherogenic burden as experienced by the reduction in apoB or apoB/apoA-I levels, but not by LDL-C in healthy middle-aged men 77. In terms of dietary fat control, it is recommended to avoid any intake of trans fatty acids, and the intake of saturated fat is supposed to be less than 10% of the total caloric intake, and should be further reduced in the presence of hypercholesterolemia (<7% of energy) 69. Compared with high MUFA (oleic acid) diets, the high saturated fat in butter or cream has a worse overall effect on blood lipids; the overall effect of marine omega-3 EPA and DHA on blood lipids seems to be protective, while omega-6 polyunsaturated fatty acids may increase the risk of coronary heart disease 78. Dietary fiber (especially soluble fiber) has the effect of lowering cholesterol, and the adverse effects of high carbohydrate diet on other lipoproteins should be minimized 79. Lifestyle intervention is very important, because in addition to lowering lipids, it also has an additional benefit on enhancing immunity. A case report has shown that a patient with Hodgkin's lymphoma who relapses with COVID-19 during chemotherapy may be the result of a weakened immune system 80.

#### Lipid-lowering drug therapy

If lifestyle intervention fails to improve hyperlipidemia, then lipid-lowering medications are needed. Dyslipidemia is very closely related to inflammation in many pathological conditions, for example, dyslipidemia and inflammation are closely related in their effects on atherosclerosis 81. The results of a meta-analysis suggest that in clinical practice, the anti-inflammatory effects of most LDL lowering therapies are related to the magnitude of change in LDL 82. There are many studies and discussions about whether COVID-19 patients should lower lipids. At present, more opinions are that statin therapy should be continued in COVID-19 patients because patients can benefit from it 83-85. For patients who have not reached the LDL-C treatment goals with high-intensity statins, and patients who are intolerant to statins, ezetimibe and PCSK9 (Proprotein convertase subtilisin/kexin type 9) inhibitors are recommended for treatment 86.

When using medications, not only should pay attention to the improvement of blood lipids, but also the possible toxic and side effects of lipid-lowering treatment 87. Once signs of liver and kidney damage and other adverse reactions appear, the use of lipid-lowering drugs should be suspended in time. Remdesivir and some statins (atorvastatin, simvastatin, and lovastatin) are metabolized in the same way, so simultaneous use should be avoided; tocilizumab interferes with the metabolic pathway of statins, so it is recommended to temporarily stop statin treatment when using tocilizumab 88. The expert team from HEART UK has summarized in detail the possible interactions between drugs used to treat COVID-19 infection and lipid-lowering drugs, as well as drug recommendations 88, which has great reference value in the management of COVID-19 patients' lipid lowering.

Both statins and PCSK9 inhibitors have been shown to have additional anti-inflammatory effects, which can reduce inflammation, oxidative stress and improve vascular inflammation, which is also one of the reasons why lipid-lowering therapy should be retained in COVID-19 patients 83. Statins refer to 3-hydroxy-3-methylglutaryl coenzyme A (HMG-CoA) reductase inhibitors as the most commonly used lipid-lowering agents and the most effective hypolipidemic compounds for reducing mortality in patients with coronary heart disease 89. HMG-CoA reductase produces mevalonate and is the rate limiting enzyme for hepatic cholesterol biosynthesis, which is competitively and reversibly inhibited by statins through both the lactone ring and the side chain, aiding in its binding to the active site of the enzyme 90. The anti-inflammatory effects of statins, whether through the clearance of proinflammatory modified LDL from the arterial wall, or through the inherent non LDL anti-inflammatory properties of isoprenoids, are well known 91.

Statins can reduce CRP in several ways: reducing LDL-C while reducing the production of ox- LDL, thereby reducing inflammation 92; reducing Rac-1 geranylgeranylation to reduce IL-6-induced serine phosphorylation of the transcription factor STAT3; inhibiting the expression of vascular cell adhesion molecule-1, intercellular adhesion molecule-1, and E-selectin; increasing the rate of CRP catabolism 93. In addition, statins may also serve anti-inflammatory purposes through their effects on endothelial NO (nitric oxide). Statins can negatively regulate endothelial nitric oxide synthase (eNOS) expression by altering NO release through multiple mechanisms, such as inhibiting the production of mevalonate and important isoprenoid intermediates, thereby preventing the prenylation of the small GTPase Rho 94. NO inhibits NF-κB by inducing and stabilizing I-κBα, thereby exerting anti-inflammatory effects 95. One study investigated whether statin treatment in the population was associated with differences in oxidative generated nucleotide damage and chronic inflammation, and the relationship between nucleotide damage and chronic inflammation, drawing from the results that statin users had a lower burden of oxidative generated DNA damage and inflammation compared with nonusers 96.

The anti-inflammatory versus antioxidant effects exerted by statins in the clinic suggested to us that it might also play the same role in COVID-19 to mitigate part of the adverse effects resulting from inflammation. Statins have previously been shown to be promising in a variety of viral infections, such as avian, H1N1 pandemic 97-99. Statins have anti-inflammatory and antithrombotic effects, including enhancing ACE2 expression, and in addition statins may also improve endothelial function in patients with COVID-19 infection, and these effects suggest that statins have potential as adjunctive therapy to attenuate endothelial dysfunction and dysregulated inflammation in covid-19-infected patients 100. There is literature discussing in greater detail the possible underlying molecular mechanisms involved in the action of statins in COVID-19: SARS-CoV-2 enters cells by binding to ACE2. Once inside the cell, SARS-CoV-2 causes ACE2 downregulation, which reduces its conversion to angiotensin mediated protection by angiotensin II. Statins upregulate ACE2 98, 99, 101, 102. It is also known that the coronavirus induces a pro-inflammatory host response through the activation of the TLR-MYD88-NF-κB pathway, which can be inhibited by statins. Finally, statins can also reduce viral entry by depleting the cholesterol content of the cell membrane, but this effect remains controversial 103.

In terms of lipid lowering, new therapies with PCSK9 inhibitors that lower LDL to a greater extent have led to new breakthroughs in the treatment of atherosclerotic cardiovascular disease 104. PCSK9 degrades low-density lipoprotein cholesterol (LDL) receptors, which in turn elevate serum LDL cholesterol. Clinical trials have shown that inhibition of PCSK9 is effective in lowering LDL cholesterol levels and reducing cardiovascular events 105. Recent studies have shown that PCSK9 is involved in the inflammatory pathway of atherosclerosis. Although trials with PCSK9 inhibitors have not shown any alterations in plasma C-reactive protein levels, there is accumulating evidence of a reduced inflammatory response in the arterial wall, which may attenuate atherosclerotic plaque development beyond the established LDL lowering effect of PCSK9 inhibitors 106. In conclusion, the available human studies show beneficial effects of statins and PCSK9 inhibitors on pneumonia and sepsis. These agents may act as immunomodulators in COVID-19 and prevent major complications such as acute respiratory distress syndrome and cytokine release syndrome 107.

#### Lipid apheresis

Simply put, lipoprotein apheresis is a therapy that filters out some lipid components in the blood to achieve the purpose of lowering blood lipids 108. Since it was first introduced, it has been the “last resort” to treat uncontrollable dyslipidemia 109. When neither diet nor medication can improve the hyperlipidemia, lipoprotein apheresis technology is a better choice. It works well but is expensive and time-consuming. In particular, for patients with familial hypercholesterolemia, lipoprotein apheresis technology is more widely used. Moreover, patients with familial hypercholesterolemia (FH) have a higher risk of CVD than normal people regardless of their age, so FH patients will have an increased risk of experiencing a severe course of COVID-19 86. Lipoprotein apheresis is currently the only truly safe and effective treatment for homozygous FH, and it is recommended that children with homozygous FH should consider lipoprotein apheresis from the age of 6 to achieve early control of cholesterol and reduce cardiovascular risk 110, 111. Lipoprotein apheresis is usually carried out once 1-2 weeks. If there is no way to achieve it, it can be postponed for two weeks with intensive lipid-lowering treatment and close monitoring of symptoms 86.

### The goal of lowering lipid

For COVID-19 patients, there is currently no study to determine the blood LDL-C cutoff value that is beneficial to the prognosis of SARS-Cov-2 infection. Therefore, the current lipid-lowering goals of COVID-19 patients are still the same as those of the non-COVID-19 population, that is, starting from reducing the risk of cardiovascular events and other complications, the treatment target value of LDL-C is drawn up according to different risk groups. As mentioned above, the current guidelines recommend reducing the LDL-C of low-risk and intermediate-risk patients, high-risk patients, and very high-risk patients to 115 mg/dl (3 mmol/l), 100 mg/dl (2.6 mmol/l) and 70 mg/dl (1.8 mmol/l), respectively 71. Even for people with low CVD risk, the LDL-C level should be maintained at a low level for a long time (LDL-C ≤ 115 mg/dL [3 mmol/L]) 112. Studies have shown that patients with a higher baseline LDL-C level benefit more from lipid-lowering therapy^113^. When the baseline LDL-C level is less than 100 mg/dL, there is no correlation between the reduction in LDL-C and the reduction in the risk of total mortality and cardiovascular mortality 113. It is worth noting that most of the lipid-lowering goals set the upper limit of LDL-C without mentioning the lower limit, mainly because the lipid-lowering ability of lipid-lowering drugs before cannot reduce the absolute value of LDL-C to a very low level, and the safety of very low cholesterol has not been evaluated 114. It is currently believed that LDL-C reaching 40-50 mg/dL seems to be safe, while the data below 25 mg/dL is limited, and more in-depth research and post-mortem analysis are needed in the future 115.

But for COVID-19 patients, cholesterol is not as low as possible. Studies have shown that in COVID-19 patients, low LDL-c serum levels (LDL-c ≤69 mg/dl) are independently associated with higher 30-day mortality 116. For patients with severe symptoms of COVID-19 who cannot take oral medications in a serious condition, lipid-lowering drugs may be temporarily discontinued 88. And some studies have shown that COVID-19 patients will have hypolipemia as early as when the symptoms are mild, and the degree of hypolipemia is positively correlated with the severity of the disease 64, 117. The current speculation is that the decrease in lipid levels is probably caused by SARS-COV-2 infection. The possible mechanisms include liver damage, acute inflammation, increased free radicals, and changes in vascular permeability 64. Compared with patients with high LDL-C levels, patients with low LDL-C levels are more likely to have immune and inflammatory dysfunction, renal dysfunction, liver dysfunction, and cardiac insufficiency on admission 118. It is still uncertain whether the secondary hypocholesterolemia of COVID-19 patients will participate in other pathophysiological mechanisms that are not conducive to the prognosis 119, so the lipid-lowering goal for COVID-19 patients may not be as low as possible. In particular, when COVID-19 patients are combined with other diseases related to hypoproteinemia, such as tuberculosis 120, 121, careful consideration should be given to whether to carry out lipid-lowering treatment. A recent study with a large sample size showed that in the Chinese population, low LDL-C levels are associated with an increased risk of all-cause, CVD, ischemic stroke, hemorrhagic stroke, and cancer mortality 122. The safety of low LDL-C needs further research.

## Conclusion

Cholesterol in the macrophages of CVD patients (as ox-LDL and CCs) activates NLRP3 and promotes the development of atherosclerosis, which also induces a systemic inflammatory state that provides favorable conditions for SARS-CoV-2 infection and indirectly leads to a poor prognosis in COVID-19 patients. Cholesterol can also directly affect the entry of viruses into host cells. First, *in vitro* studies showed that membrane cholesterol can increase the number of endocytic entry points on the host cell membrane and transport ACE2 to the endocytic entry points, thereby increasing the entry of pseudoSARS-CoV-2. Second, previous studies showed that viral fusion proteins or spikes have receptors cholesterol, and recent studies on SARS-CoV-2 have shown that spikes require cholesterol to enter cells. In addition, SR-B1 can help the spike's S1 subunit to bind to HDL cholesterol in the blood and promote the entry of SARS-CoV-2 into the host cells. However, more detailed molecular mechanisms remain to be studied. It is currently recommended to maintain the original lipid-lowering treatment for COVID-19, but very low LDL-C levels are not recommended. Specific lipid-lowering programs and precautions can refer to the current guidelines.

## Figures and Tables

**Table 1:** The critical value of the lipid balance of each component in a normal blood test

Category	U.S. and some other countries	Canada and most of Europe
TC	<200 mg/dL	<5.18 mmol/L
LDL-C	<100 mg/dL	<2.6 mmol/L
HDL-C	>60 mg/dL	>1.5 mmol/L
TG	<150 mg/dL	<1.7 mmol/L

TC: total cholesterol; LDL-C: LDL cholesterol; HDL-C: HDL cholesterol; TG: Triglycerides.
